# Global research trends in embryonic mosaicism and mosaic embryo transfer: a bibliometric and science-mapping analysis

**DOI:** 10.3389/fmed.2026.1860968

**Published:** 2026-08-12

**Authors:** Mustafa Akşar, Sezin Eda Karsli, Özgecan Uçyildiz Gülmez

**Affiliations:** Department of Obstetrics and Gynecology, Hacettepe University Faculty of Medicine, Ankara, Türkiye

**Keywords:** bibliometric analysis, embryonic mosaicism, mosaic embryo transfer, next-generation sequencing, preimplantation genetic testing for aneuploidy (PGT-A), science mapping

## Abstract

**Background:**

Embryonic mosaicism has emerged as a clinically significant topic in assisted reproductive technologies following the widespread adoption of preimplantation genetic testing for aneuploidy (PGT-A). This study aimed to characterize the global research landscape, identify major contributors, and delineate emerging thematic trends in mosaic embryo research using bibliometric and science mapping approaches.

**Methods:**

We conducted a bibliometric and science mapping analysis of original research articles and reviews published between 1990 and 2025 that focused on embryonic mosaicism, mosaic embryos, mosaic embryo transfer, or PGT-A-related mosaicism in reproductive medicine. Publication trends, citation impact, collaboration networks, and thematic evolution were analyzed using Bibliometrix and VOSviewer.

**Results:**

We included 394 eligible original research and review articles on embryonic mosaicism, mosaic embryo transfer, or PGT-A-related mosaicism in reproductive medicine, revealing a marked acceleration in research activity after 2015. The United States led in both publication volume (*n* = 92 corresponding-author publications) and total citation impact (4,406 citations), while the Netherlands demonstrated the highest citation efficiency (105.3 citations/publication). The University of Kent led in institutional productivity (16 publications), while the University of Oxford demonstrated the highest per-paper citation impact (112.1 citations/publication). Three thematic clusters—cytogenetic characterization, NGS-based genomic diagnostics, and clinical outcomes—were consistently identified across keyword co-occurrence, co-citation, and bibliographic coupling analyses, with recent trends toward non-invasive PGT-A and artificial intelligence signaling an emerging fourth domain.

**Conclusion:**

This bibliometric analysis of 394 publications spanning 36 years demonstrates sustained, accelerating growth in research output since 2015, driven by the clinical adoption of NGS-based PGT-A and the subsequent dissemination of major professional society guidelines. The field has evolved from cytogenetic characterization to a clinically integrated discipline that directly informs embryo selection and IVF decision-making. Significant knowledge gaps persist regarding mosaicism thresholds, self-correction mechanisms, and long-term neonatal outcomes, underscoring the need for prospective registries and standardized reporting frameworks.

## Introduction

Embryonic mosaicism refers to the presence of two or more genetically distinct cell populations within a single embryo, typically caused by mitotic errors during early development. This condition results in the coexistence of euploid and aneuploid cell lineages and is a complex biological phenomenon with important implications for embryo viability. Historically, embryos identified as mosaic were deemed abnormal and excluded from transfer during *in vitro* fertilization (IVF) treatments. However, advances in genomic technologies have challenged this traditional perspective and prompted a reevaluation of the biological and clinical significance of embryonic mosaicism.

The widespread adoption of next-generation sequencing (NGS) technologies in preimplantation genetic testing for aneuploidy (PGT-A) has greatly improved the resolution and sensitivity of chromosomal screening in human embryos. Consequently, mosaic chromosomal abnormalities are now detected more frequently than with earlier diagnostic methods such as fluorescence *in situ* hybridization (FISH) or array comparative genomic hybridization (aCGH); it should be emphasized that this reflects the greater analytical sensitivity of NGS in identifying intermediate copy-number states rather than a true increase in the underlying biological prevalence of mosaicism (1, 25). This technological progress has led to the recognition of mosaic embryos as an intermediate category between euploid and aneuploid embryos, posing new challenges for embryo selection strategies and clinical decision-making in assisted reproductive technologies. The diagnostic history of the field mirrors this technological progression: early cytogenetic detection relied on FISH, which permitted only a limited number of chromosomes to be assessed per cell; the subsequent adoption of aCGH enabled genome-wide copy-number analysis, and the more recent transition to NGS provided the quantitative resolution needed to define intermediate—mosaic—copy-number states (23, 24). This progression, together with the publication of major professional guidelines—the PGDIS position statements, the ESHRE good-practice recommendations, and the ASRM committee opinion—has reshaped how mosaic findings are interpreted and clinically managed. Importantly, mosaic embryos occupy a uniquely ambiguous clinical position: because they lie at the boundary between euploid and aneuploid embryos, their reproductive potential cannot be predicted with certainty, and both their interpretation and the associated patient counseling remain particularly challenging—a clinical ambiguity that provides the central rationale for a dedicated bibliometric analysis of this field.

A significant milestone in this field occurred when clinical reports showed that selected mosaic embryos could lead to successful pregnancies and healthy live births (2). These findings sparked ongoing scientific debate about the reproductive potential and safety of mosaic embryo transfer and highlighted the need for improved risk-stratification strategies. In response, professional societies—including the Preimplantation Genetic Diagnosis International Society (PGDIS), the American Society for Reproductive Medicine (ASRM), and the European Society of Human Reproduction and Embryology (ESHRE)—have issued recommendations to guide the interpretation and clinical management of mosaic embryo findings.

Although mosaic embryo transfer is increasingly accepted in clinical practice, three critical questions remain unresolved: the mosaicism threshold at which transfer risk becomes clinically unacceptable, the biological mechanisms underlying *in vivo* self-correction, and the long-term health outcomes of children born from mosaic embryo transfers. These unanswered questions have driven a substantial rise in research activity over the past decade, fueled by technological advances and the growing clinical demand for evidence-based embryo selection strategies.

Along with this rapid expansion of the literature, bibliometric analysis has become a valuable instrument for systematically evaluating scientific productivity, collaboration structures, and emerging research themes within a field. Despite the growth of the mosaic embryo literature, systematic evaluations of this domain remain limited. Existing bibliometric studies on preimplantation genetic testing have addressed PGT-A or related topics without characterizing the structure, collaborative architecture, or thematic evolution of mosaic embryo research as a distinct scientific domain. No prior study has integrated publication trend analysis, network visualization, and keyword mapping to track the field's development from the cytogenetic era through the current NGS period. Furthermore, the post-guideline landscape—shaped by the PGDIS 2021 position statement, ESHRE 2022 survey, and ASRM 2023 committee opinion—has not been comprehensively analyzed, despite these documents having fundamentally reshaped clinical practice. This study addresses these gaps by conducting a comprehensive bibliometric and science-mapping analysis of global research on embryonic mosaicism and mosaic embryo transfer from 1990 to 2025 to identify publication trends, leading contributors, influential journals, and the longitudinal evolution of key research themes.

## Materials and methods

### Study design

This study was conducted in accordance with established bibliometric research frameworks and reporting standards to assess global publication trends, key research contributors, and collaboration networks in embryonic mosaicism and mosaic embryo transfer research. Bibliometric methods quantify the growth of a research field by analyzing publication outputs, citation patterns, co-authorship networks, and keyword relationships.

### Data source and search strategy

A comprehensive literature search was performed across three electronic databases: Web of Science Core Collection (WoSCC), Scopus, and PubMed. Searches were conducted on March 31, 2026, covering the period from January 1, 1990, to December 31, 2025. No language or document-type restrictions were applied at the initial retrieval stage; these filters were applied during the eligibility screening phase. Publications indexed in 2024 and 2025 may be subject to an indexing lag—a recognized limitation in bibliometric studies conducted within 12–24 months of the reference year—and citation counts for these cohorts should therefore be interpreted with caution.

A topic-based search was conducted using a revised, logically structured Boolean query to ensure thematic specificity and consistent retrieval. Mosaicism-related terms were grouped within a single OR cluster and combined with a reproductive medicine context cluster via the AND operator, thereby eliminating the operator-precedence ambiguity present in preliminary search iterations. The following query was applied in WoSCC:

*TS* = ((“mosaic embryo transfer” OR “embryo mosaicism” OR “mosaic embryo^*^” OR “mosaic blastocyst” OR “blastocyst mosaicism” OR “chromosomal mosaicism”) AND (“preimplantation genetic testing” OR “PGT-A” OR “preimplantation genetic screening” OR “PGS” OR “in vitro fertilization” OR “IVF” OR “assisted reproductive technolog^*^”* OR “ART”))*

Equivalent queries were applied in Scopus and PubMed using database-specific field tags.

### Eligibility criteria

#### Inclusion criteria

1. Publications focusing on embryonic mosaicism, mosaic embryos, mosaic embryo transfer, or mosaicism detected during PGT-A.
2. Publications related to reproductive medicine, genetics, embryology, obstetrics, or gynecology.
3. Original research articles and review articles.
4. Articles published from 1990 to 2025.

#### Exclusion criteria

1. Editorials, letters, meeting abstracts, corrections, and conference proceedings.
2. Publications unrelated to reproductive medicine, embryology, or reproductive genetics.

### Study selection process

**Figure 1:**
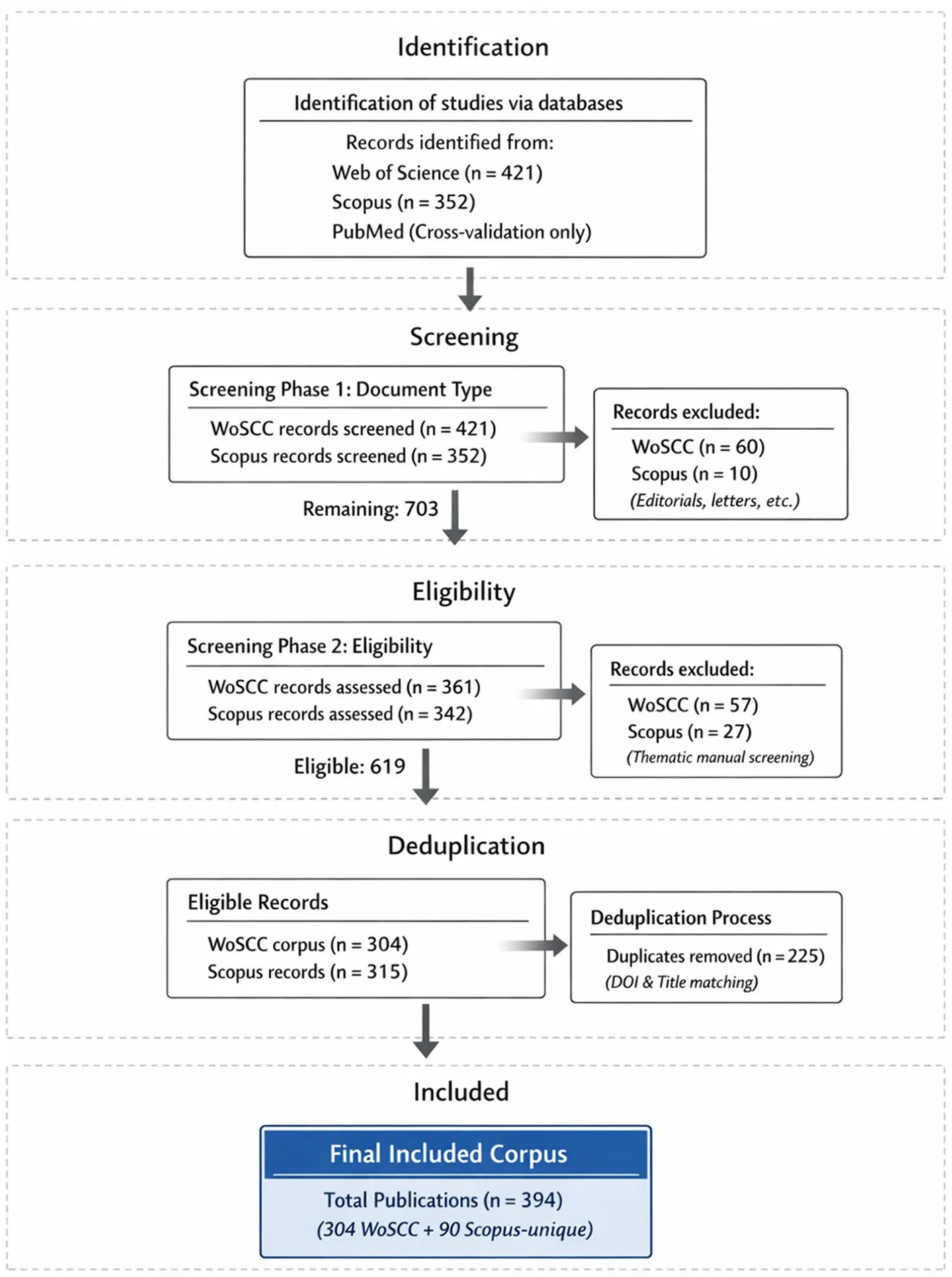
PRISMA flow diagram of the study selection process. The diagram illustrates the two-stage selection process applied to retrieve publications on embryonic mosaicism and mosaic embryo transfer. In stage 1, WoSCC records underwent a full PRISMA-based workflow: 421 records were identified, 60 excluded based on document type, and 57 excluded following manual thematic screening, yielding 304 publications. In stage 2, 90 Scopus-unique records were incorporated after deduplication. A concurrent PubMed search (*n* = 203) confirmed 88.7% corpus coverage and contributed no additional records, serving solely as a cross-validation source.

The study selection process used a two-stage screening workflow—document-type exclusion followed by independent thematic assessment by two reviewers (MA and SEK)—applied to WoSCC and Scopus in parallel, with PubMed used as a coverage-validation source. The Boolean search retrieved 421 records from WoSCC and 352 from Scopus; after document-type and thematic screening, the two databases yielded 304 and 315 eligible publications, respectively. Records were merged and deduplicated via pairwise DOI matching, supplemented by normalized title matching when DOIs were absent, yielding a final analytical corpus of 394 publications (304 from WoSCC and 90 Scopus-unique). Inter-rater agreement was excellent at both stages (document-type κ = 0.91; thematic κ = 0.87), with disagreements resolved by consensus. The complete workflow, including stage-specific record counts, is presented in the PRISMA flow diagram (Figure 1).

A parallel PubMed search (203 records) confirmed the completeness of this corpus: 180 records (88.7%) were already represented, and most of the 23 PubMed-unique records were ineligible under the pre-specified criteria or were non-English. PubMed served as a validation resource rather than a primary source because it does not export the structured cited-reference lists and standardized affiliation and citation fields required for co-citation, bibliographic coupling, and citation impact analyses; incorporating its records directly would have introduced entries lacking the fields on which these analyses depend. This cross-validation confirms that the merged WoSCC–Scopus dataset provides a comprehensive and representative base for the analysis.

### Data extraction and preprocessing

Complete bibliographic records were exported from WoSCC (plain text) and Scopus (Excel) and merged after DOI- and title-based deduplication to generate the primary analytical corpus of 394 publications. PubMed records (MEDLINE format) were exported separately and used only for coverage validation, not for citation-based or network analyses. Extracted variables included authors, titles, source journals, publication years, author keywords, institutional affiliations, countries, citation counts, and funding information. Preprocessing steps were applied to the WoSCC and Scopus analytical records, including removing duplicate records, standardizing author name variants, harmonizing institutional affiliations, and consolidating synonymous keywords. For keyword normalization, semantically equivalent terms (e.g., “PGT-A” and “preimplantation genetic testing for aneuploidy”) were unified using a thesaurus-based approach within VOSviewer.

### Bibliometric and science-mapping analysis

Bibliometric and science-mapping analyses were conducted using VOSviewer (version 1.6.20; Van Eck and Waltman, Leiden University) and the Bibliometrix R package. Citation impact was assessed through total citations, average citations per publication, and h-index. Network analyses characterized collaboration patterns among countries, institutions, and authors and mapped the field's intellectual structure through co-citation analysis. Keyword co-occurrence analysis identified dominant themes and emerging research topics, while bibliographic coupling delineated related research clusters based on shared reference profiles. Visualization employed association-strength normalization to minimize the influence of highly prolific nodes, and cluster assignment used VOSviewer's modularity-based algorithm. All counts were computed using the full-counting method, which assigns equal weight to all contributing authors and institutions, ensuring transparency and minimizing attribution bias. All analyses followed established bibliometric and science mapping guidelines (3).

### Threshold settings

To enhance interpretability and minimize noise in bibliometric network maps, the following minimum thresholds were applied: keyword occurrence ≥ 5; country publication count ≥ 5; and institutional publication count ≥ 5. These values were determined based on commonly accepted practices in bibliometric research to balance data inclusiveness and network interpretability (3). To assess robustness, a sensitivity analysis was conducted using alternative thresholds of *n* ≥ 3 and *n* ≥ 10.

### Temporal trend analysis

To examine the temporal association between NGS adoption and literature growth, annual publication counts and annual NGS-related publication counts were analyzed over the 1990–2025 period. NGS-related publications were defined as records containing “NGS” or “next-generation sequencing” in title, abstract, or keyword fields. It should be noted that the keyword fields used to classify NGS-related publications partially overlap with those analyzed in the keyword co-occurrence network; however, the two analyses operate at different units of analysis—publications vs. keyword nodes—and are therefore treated as complementary rather than redundant. Monotonic trends were assessed using the Mann-Kendall test; the association between annual NGS-related and total output was evaluated using Spearman's rank correlation for the 2014–2025 expansion subperiod. A two-sided *p*-value < 0.05 was considered statistically significant.

### Comparator publication trend analysis

To contextualize whether the increase in mosaic-embryo research exceeded the broader growth of assisted-reproduction literature, we retrieved annual PubMed publication counts for two comparator fields from 1990 to 2025: IVF/ART and PGT/PGS. The IVF/ART comparator included title/abstract terms for “*in vitro* fertilization,” “IVF,” and “assisted reproductive technology/technologies.” In contrast, the PGT/PGS comparator included “preimplantation genetic testing,” “PGT-A,” “preimplantation genetic screening,” “PGS,” and “preimplantation genetic diagnosis.” Searches were conducted on the same date as the primary search. Annual counts and the proportional contribution of mosaic-embryo publications to each comparator field were analyzed descriptively. PubMed was selected as the comparator source because it provides a single, stable, and freely reproducible denominator for the broad IVF/ART and PGT/PGS literatures across the full 1990–2025 window, whereas assembling equivalent whole-field counts from WoSCC and Scopus would require large-scale de-duplication that is neither necessary nor appropriate for a descriptive denominator. Because the comparator counts were therefore obtained from PubMed while the primary corpus was built from WoSCC and Scopus, and because the analysis compares proportional growth trajectories rather than absolute record counts, this comparison should be interpreted as an assessment of relative field-level growth rather than of record-level equivalence; any modest cross-database coverage differences apply similarly to the numerator and denominator fields and would not be expected to alter the direction or approximate magnitude of the observed disproportionate growth. Annual comparator counts are presented in Supplementary Table S1.

### Commercial affiliation and funding assessment

To characterize the involvement of commercial interests in this research domain, each of the 394 publications in the analytical corpus was assessed for a commercial link using a pre-specified, hierarchical classification applied by two reviewers (MA and SEK). A publication was classified as commercially linked if it satisfied at least one of the following criteria: (i) one or more authors listed an affiliation with a commercial provider of PGT-A, reproductive genetic testing, or IVF-network laboratory services in the byline affiliation fields; (ii) the funding or acknowledgment statement declared financial support from such a provider; or (iii) the declared conflict-of-interest statement disclosed employment, a founding or executive role, equity, consultancy, or a directly relevant collaborative relationship with such a provider. Publications meeting none of these criteria were classified as non-commercial.

Two nested indicators were derived from this assessment and are reported separately to avoid conflation. The narrower indicator, declared commercial funding, counted only publications meeting criterion (ii) and was used in the author-level Results (“Commercial Affiliations Among Leading Contributors”). The broader indicator, industry affiliation, counted publications meeting any of criteria (i)–(iii) and was used in the corpus-level Discussion analysis. The two indicators are therefore not interchangeable, and the difference between them reflects the well-recognized observation that industry involvement in this field is more frequently expressed through author affiliation and disclosed relationships than through declared study funding.

Affiliation, funding, and conflict-of-interest information was extracted primarily from the structured WoSCC and Scopus records (affiliation and funding-acknowledgment fields) and, where these were incomplete, cross-checked against the conflict-of-interest and funding statements of the corresponding full-text article. Organizations were classified as commercial providers when they were for-profit entities offering PGT-A, preimplantation genetic testing, or associated laboratory or IVF-network services; academic departments, non-profit foundations, and public hospitals were not counted as commercial affiliations even where they conducted PGT-A clinically. Author-level commercial relationships (Supplementary Table S5) were verified by cross-referencing the disambiguated Scopus Author ID with the affiliation and disclosure information described above. Discrepancies in classification were resolved by consensus.

This assessment is explicitly descriptive. It characterizes the field's authorship and funding structure at the corpus level. It is not intended as an evaluation of individual investigators, of the presence or absence of bias in any specific study, or of the scientific validity of commercially affiliated research. Because the classification relies on information disclosed within the publications themselves, undisclosed relationships cannot be captured, and the reported proportions should be regarded as conservative lower-bound estimates.

### Outcome measures

Primary outcomes included: temporal evolution of publication productivity; leading countries, institutions, journals, and authors; citation impact indicators (total citations, h-index); international collaboration patterns; and thematic evolution based on keyword co-occurrence, co-citation, and bibliographic coupling analyses. Secondary outcomes included identification of research gaps and emerging translational priorities relevant to mosaic embryo transfer.

## Results

### Publication output and trends over time

**Figure 2:**
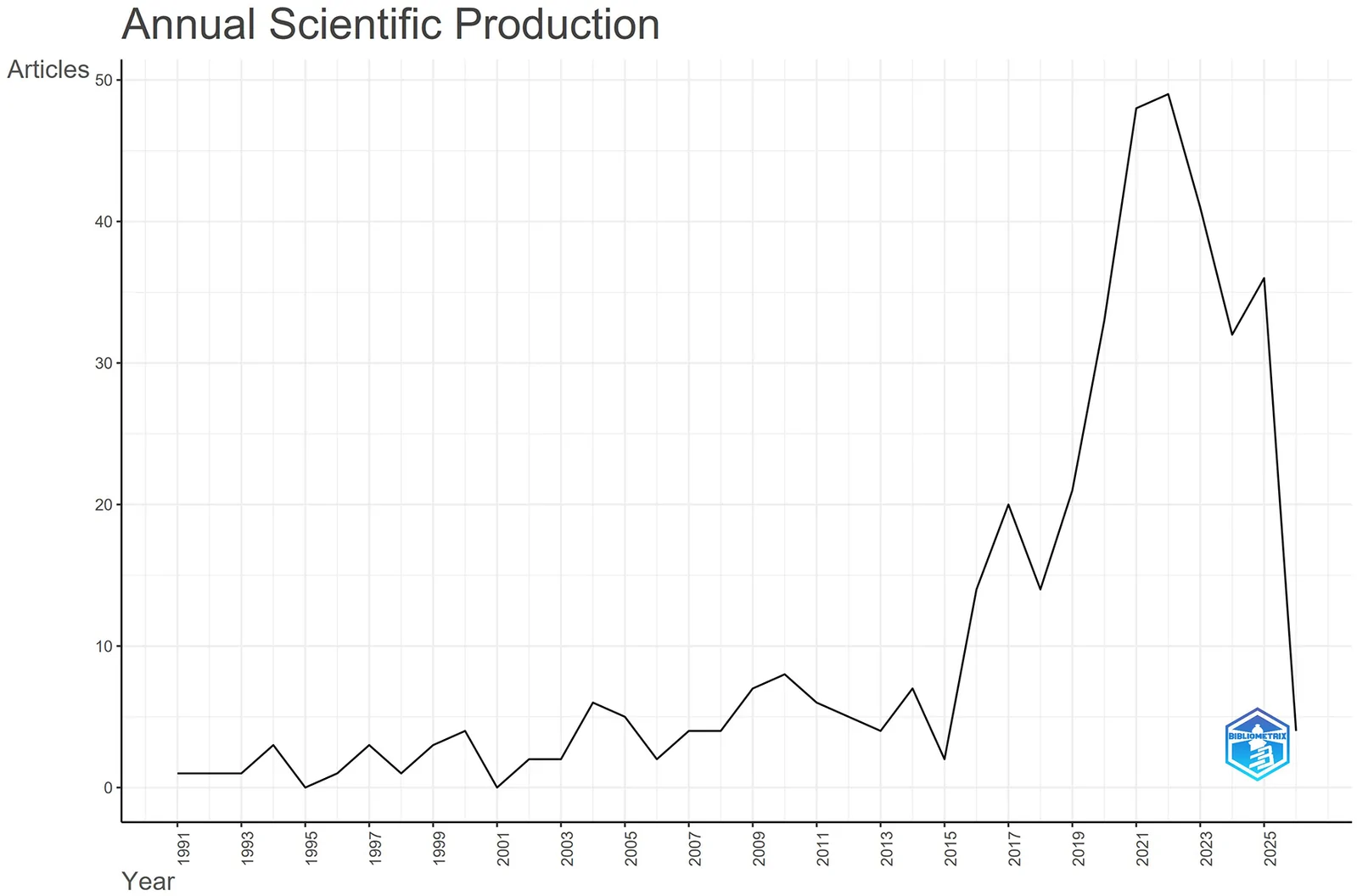
Annual publication trends in embryonic mosaicism and mosaic embryo research (1990–2025). The annual number of publications remained low until 2015, then increased substantially, coinciding with the widespread adoption of NGS-based PGT-A. This figure were generated using the Bibliometrix R package.

Across the three databases, records were eligible if they addressed embryonic mosaicism, mosaic embryos, or mosaicism detected during PGT-A within a reproductive-medicine context and were original research articles or reviews published between 1990 and 2025; editorials, letters, meeting abstracts, corrections, and conference proceedings were excluded. Of the records identified in the primary databases (421 in WoSCC and 352 in Scopus), 394 met these criteria after document-type and thematic screening and DOI-based deduplication, and were carried forward for analysis. The merged WoSCC–Scopus selection process yielded a final corpus of 394 publications, comprising 317 articles (80.5%)—including 312 original research articles, three article/proceedings papers, and 2 article/book chapters—and 77 reviews (19.5%)—comprising 76 reviews and 1 review/book chapter — drawn from 126 distinct source journals. The study selection process is summarized in the PRISMA flow diagram (Figure 1). Annual publication output remained low throughout the 1990–2014 period, generally in the single digits (typically fewer than 10, with a maximum of 8 in 2010; Figure 2). A marked inflection occurred in 2015 and accelerated sharply in 2016 (*n* = 14), coinciding with the clinical introduction of NGS-based PGT-A and the first reports of successful live births following mosaic embryo transfer. Output continued to rise, peaking in 2022 (*n* = 49; 12.4% of the total dataset), followed by a modest decline in 2023 (*n* = 41) and 2024 (*n* = 32; 8.2%). As of the search date (March 31, 2026), 36 publications had been indexed for 2025; this figure reflects partial-year indexing coverage and should be interpreted descriptively.

Trend analysis confirmed a significant, monotonic increase in overall publication output over the study period (Mann–Kendall tau = 0.689, *p* < 0.001). NGS-related publications increased in parallel (tau = 0.444, *p* < 0.001), with particularly strong growth between 2014 and 2025 (tau = 0.606, *p* = 0.007). During this expansion phase, annual NGS-related output was strongly correlated with total annual output (Spearman's rho = 0.843, *p* < 0.001), consistent with NGS adoption as a principal driver of field growth. A secondary acceleration between 2021 and 2023 coincides with the dissemination of updated professional society recommendations—specifically, the PGDIS 2021 position statement (4), the ESHRE 2022 survey (5), and the ASRM 2023 committee opinion (6)—which collectively operationalized a risk-stratified framework for mosaic embryo management. To determine whether this acceleration reflected a mosaic-specific phenomenon rather than the general expansion of assisted-reproduction research, annual output in this corpus was benchmarked against two reference literatures indexed in PubMed over the same period: the broad IVF/ART literature and, as a more proximate control, the PGT-A literature. Both reference fields grew over the study window, but at markedly lower rates than those observed in mosaic-embryo research. Between 2015 and 2022, annual IVF/ART output rose by approximately 1.6-fold, and PGT-A output by approximately 2.1-fold. In contrast, mosaic-embryo output increased far more steeply, rising from single-digit annual counts before 2015 (for example, seven publications in 2014) to a peak of 49 in 2022, an approximately seven-fold increase over that interval. Correspondingly, the share of the PGT-A literature specifically addressing embryonic mosaicism rose from about 1.6% in the 1990–2010 period to about 3.8% in 2018–2025, and its share of the broader IVF/ART literature increased approximately threefold over the same periods. This nested comparison indicates that the growth documented here is not merely a reflection of the overall rise in IVF research output but represents a disproportionate, topic-specific intensification of interest in embryonic mosaicism. Because the reference literature was retrieved from PubMed, whereas the primary corpus was built from WoSCC and Scopus, these comparisons should be interpreted as relative growth trends rather than exact record-level equivalences.

### Leading countries in mosaic embryo research

**Figure 3:**
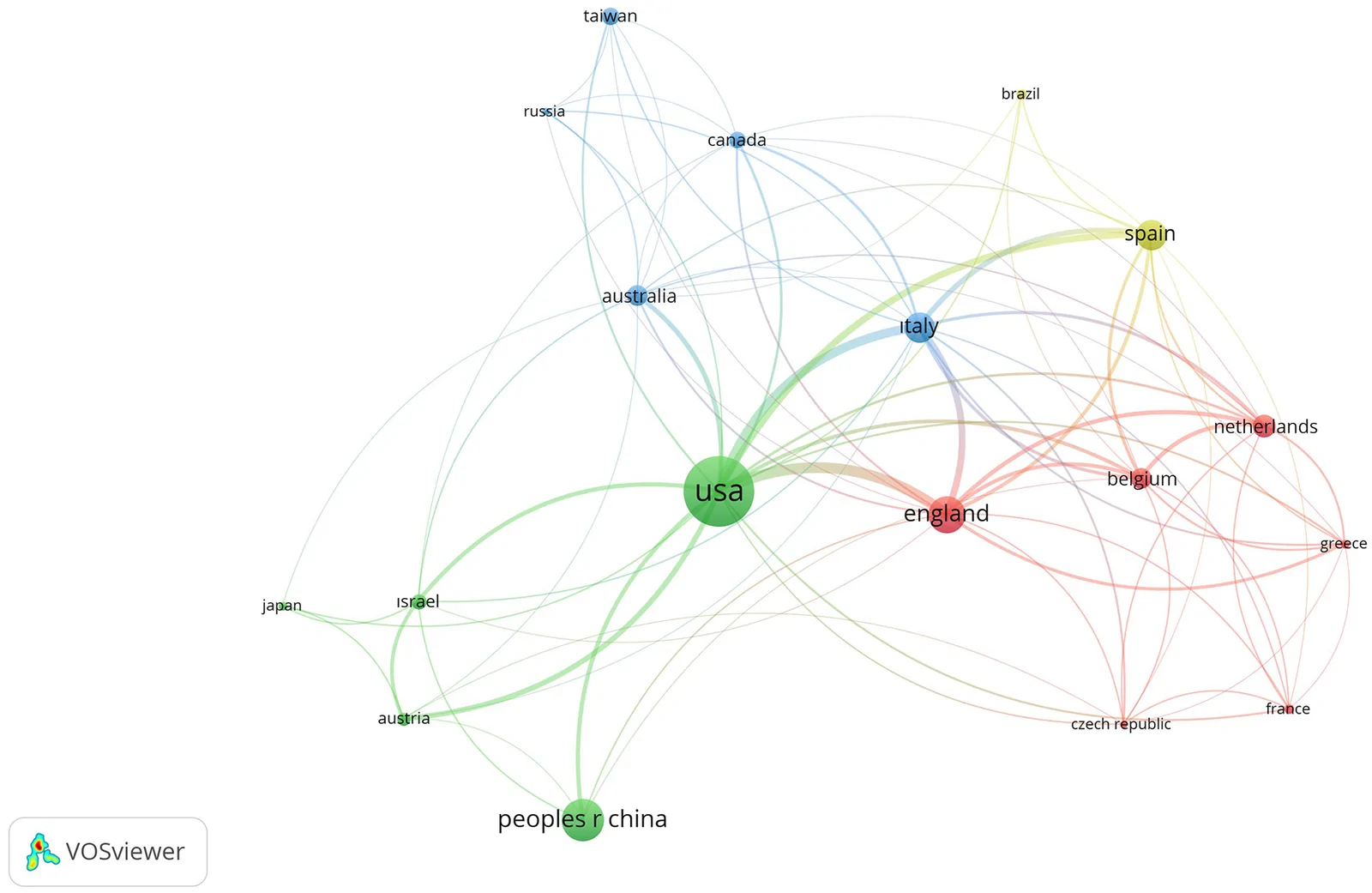
International collaboration network in embryonic mosaicism and mosaic embryo research. Network visualization generated using VOSviewer based on co-authorship relationships between countries. Node size corresponds to publication count; link thickness indicates collaboration strength. Countries with at least five publications were included. This figure were generated using VOSviewer (v1.6.20).

Publications originated from 48 countries (all-author field; 34 by corresponding author). The United States led in both productivity (*n* = 92) and total citation impact (4,406 citations), followed by China (*n* = 89), the United Kingdom, Spain, Italy, and the Netherlands. Publication volume and citation efficiency diverged markedly: the Netherlands recorded the highest per-publication impact (105.3 citations/publication) and China the lowest among leading contributors (11.6 citations/publication), the latter consistent with its low international collaboration rate (MCP = 7.9%) relative to the Netherlands and the United Kingdom. The collaboration network (Figure 3) places the United States centrally, with strong ties across Europe and East Asia. Full country-level indicators are provided in Supplementary Table S2; citation-based metrics should be interpreted with caution given differences in publication age and citation maturity.

### Leading institutions

**Figure 4:**
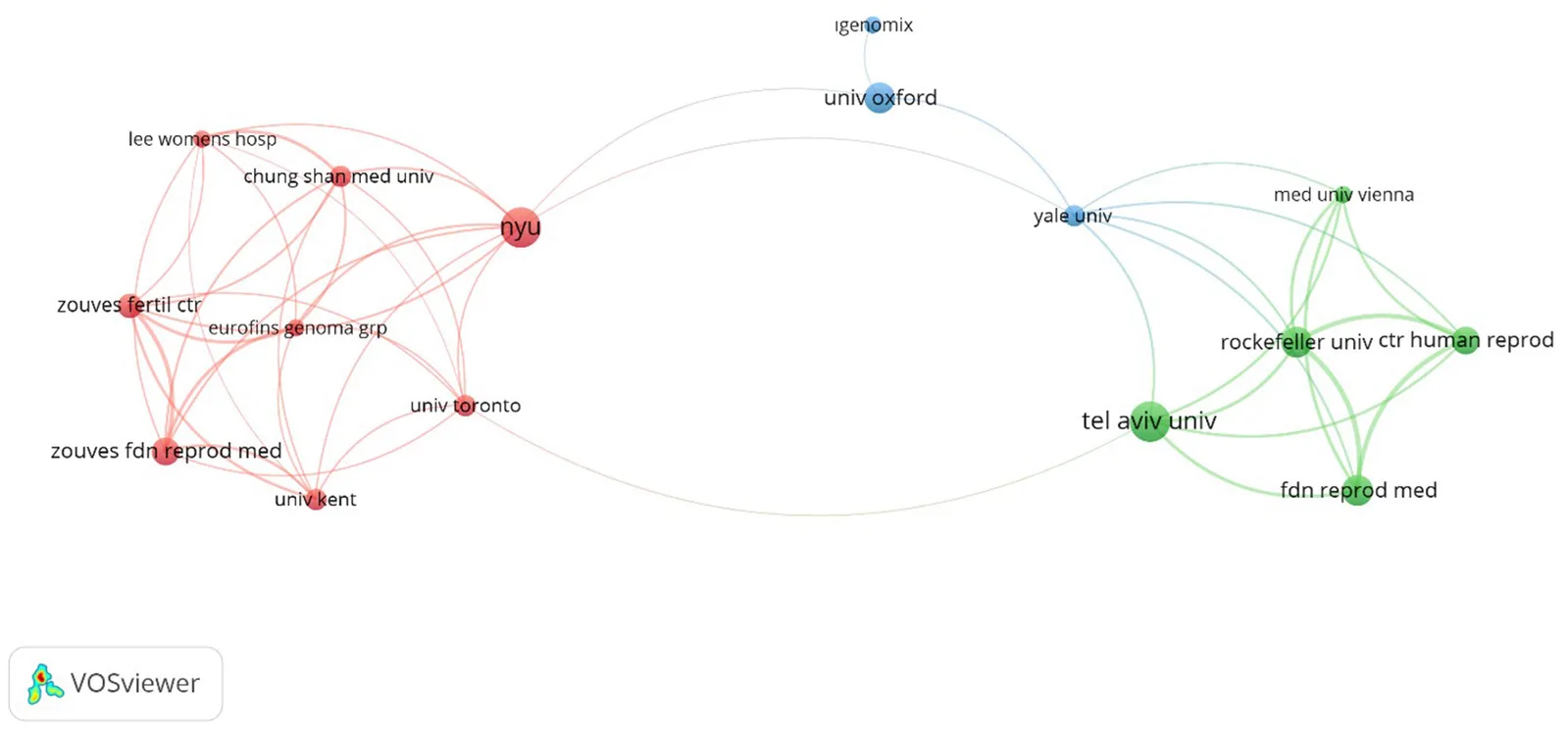
Institutional collaboration network in embryonic mosaicism and mosaic embryo research. Network map illustrating collaborative relationships among research institutions based on co-authorship data. Node size reflects publication count; colors represent collaborative clusters. Institutions with at least five publications were included. This figure were generated using VOSviewer (v1.6.20).

The University of Kent led institutional productivity (16 publications), followed by the University of London (*n* = 15), New York University and University College London (*n* = 14 each). At the institutional level, productivity and citation impact diverged: the University of Oxford recorded the highest per-paper impact (112.1 citations/publication across 9 publications). At the same time, Shanghai Jiao Tong University exhibited a purely domestic collaboration pattern (MCP = 0.0%). The institutional network (Figure 4) is dominated by cross-border collaboration among North American, Southern European, and Northern European reproductive-genetics centers. Institutions were counted at the level of the affiliation string reported in the source records; consequently, constituent colleges of federal universities (for example, University College London within the University of London system) are reported as distinct entities in accordance with standard bibliometric practice, and their counts should not be summed. Full institutional indicators are provided in Supplementary Table S3.

### Leading journals

The literature was moderately concentrated across 126 journals: three titles—the Journal of Assisted Reproduction and Genetics (*n* = 46), Fertility and Sterility (*n* = 44), and Human Reproduction (*n* = 42)—accounted for one-third of output, consistent with Bradford's core-zone structure. Publication volume and citation impact again diverged: Human Reproduction and Fertility and Sterility carried the greatest total impact (*h*-index = 28 each), while Human Reproduction Update showed the highest citation efficiency (164.3 citations/publication) on the strength of a few influential systematic reviews. The full journal ranking is provided in Supplementary Table S4.

### Highly cited publications

The 394 publications received a combined total of 13,867 citations, with a mean of 35.2 citations per publication and an overall dataset *h*-index of 66.

The most-cited publication in the dataset was Baart et al. (26), with 409 citations (an average of 20.4/year), demonstrating that milder ovarian stimulation protocols reduce the incidence of embryo aneuploidy and mosaicism in IVF. Bolton et al. (27) ranked second with 387 citations (35.2/year), providing experimental evidence, using a mouse model, that aneuploid cells are selectively depleted from the inner cell mass lineage—a mechanistic basis for embryonic self-correction. Delhanty et al. (28) ranked third with 322 citations, having introduced multicolor fluorescence *in situ* hybridization to detect chromosomal mosaicism in preimplantation embryos. Taylor et al. (29), with 317 citations (24.4/year), provided the most comprehensive pre-NGS systematic review of the origins, mechanisms, and clinical consequences of chromosomal mosaicism, and it remains a foundational reference for contemporary investigators. Mastenbroek et al. (30), with 316 citations, ranked fifth, representing a pivotal methodological landmark as the first rigorous systematic review and meta-analysis of randomized controlled trials evaluating the clinical evidence for preimplantation genetic screening. Capalbo et al. (31) ranked ninth with 217 citations (36.2/year), representing the highest annual citation rate among prospective clinical trials in the dataset, reflecting the field's demand for non-selection study designs. The 15 most-cited publications are summarized in Table 1. Beyond the six studies detailed above, the remaining table entries similarly reflect the field's principal findings: Delhanty et al. (32) provided the earliest FISH-based demonstration of chromosomal mosaicism in preimplantation embryos; van Echten-Arends et al. (33) established, through meta-analysis, that mosaicism is highly prevalent in cleavage-stage embryos; Munné et al. (7) and Fragouli et al. (34) reported that NGS-classified mosaic blastocysts can implant and produce live births at rates intermediate between euploid and aneuploid embryos; McCoy et al. (35) demonstrated selection against complex mitotic-origin aneuploidy during early development; and Spinella et al. (36) showed that clinical outcomes deteriorate as the degree of mosaicism increases. To make Table 1 more informative, its final column summarizes the study design and the principal finding or contribution of each publication, rather than providing only bibliometric information.

**Table 1:** Top 15 most cited publications within the retrieved dataset, with study design and principal findings.

Rank	First author	Year	Total citations	Avg citations/ year	Study design and key contributions DOI
1	Baart EB	2007	409	20.4	RCT (Prospective randomized controlled trial; mild vs. conventional ovarian stimulation)
					10.1093/humrep/del484
2	Bolton H	2016	387	35.2	Experimental–Animal Model Study (Mouse model of chromosome mosaicism; lineage-specific depletion of aneuploid cells in ICM lineage vs. trophectoderm; normal developmental potential confirmed)
					10.1038/ncomms11165
3	Delhanty JDA	1997	322	10.7	Observational–FISH Laboratory Study (Non-interventional; surplus cleavage embryos from PGD cycles, fertile donors)
					10.1007/s004390050443
4	Taylor TH	2014	317	24.4	Systematic Review (Narrative/systematic review of mosaicism origins, mechanisms, and clinical consequences)
					10.1093/humupd/dmu016
5	Mastenbroek S	2011	316	19.8	Systematic Review & Meta-analysis (Meta-analysis of RCTs on PGS; 9 RCTs included)
					10.1093/humupd/dmr003
6	Delhanty JD	1993	271	8.0	Observational–FISH Laboratory Study (Pioneering FISH-based detection of aneuploidy and chromosomal mosaicism in preimplantation embryos during sex determination cycles)
					10.1093/hmg/2.8.1183
7	Baart EB	2006	228	10.9	Observational–FISH Laboratory Study (PGS-based FISH analysis; surplus embryos from young IVF patients; cross-sectional)
					10.1093/humrep/dei291
8	van Echten-Arends J	2011	224	14.0	Systematic Review & Meta-analysis (36 studies; meta-analysis of chromosomal constitution data from preimplantation embryos)
					10.1093/humupd/dmr014
9	Capalbo A	2021	217	36.2	Prospective Cohort (Non-selection clinical trial; NGS-based mosaicism classification; developmental potential and live birth outcomes of mosaic blastocysts assessed prospectively without PGT-A-based de-selection; Am J Hum Genet)
					10.1016/j.ajhg.2021.11.002
10	Munné S	2017	214	23.8	Retrospective Cohort (Multi-center; NGS-based PGT-A; mosaic blastocyst outcomes vs. euploid controls)
					10.1016/j.fertnstert.2017.05.002
11	Fragouli E	2011	211	13.2	Observational–Comparative Laboratory Study (TE biopsy; blastocysts analyzed by FISH/CGH/aCGH; diagnostic accuracy evaluation)
					10.1093/humrep/deq344
12	Munné S	1997	193	6.4	Observational–Multi-center Laboratory Study (FISH analysis; donated embryos from 4 IVF centers; treatment-related mosaicism rates)
					10.1093/humrep/12.4.780
13	Fragouli E	2017	189	18.9	Retrospective Cohort (Archived TE biopsy specimens; NGS re-analysis; mosaic vs. euploid implantation/miscarriage/ongoing pregnancy)
					10.1007/s00439-017-1797-4
14	McCoy RC	2015	171	14.2	Retrospective Cohort–SNP Array Analysis (Large-scale PGS dataset; evidence of selection against complex mitotic-origin aneuploidy during preimplantation development; PLOS Genetics)
					10.1371/journal.pgen.1005601
15	Spinella F	2018	169	18.8	Retrospective Cohort (Multi-center; NGS-based PGT-A; correlation between degree of chromosomal mosaicism—low vs. high level—and clinical outcomes; Fertility and Sterility)
					10.1016/j.fertnstert.2017.09.025

Study designs are classified based on the WoSCC document type field combined with methodological review. RCT, randomized controlled trial; FISH, fluorescence *in situ* hybridization; ICM, inner cell mass; TE, trophectoderm; NGS, next-generation sequencing; PGT-A, preimplantation genetic testing for aneuploidy; aCGH, array comparative genomic hybridization; SNP, single nucleotide polymorphism.

The thematic distribution of highly cited publications confirms three dominant research trajectories: (1) the detection and prevalence of chromosomal mosaicism using successive genomic technologies (FISH, aCGH, NGS); (2) the relationship between mosaicism level and reproductive outcomes following embryo transfer; and (3) critical appraisal of the evidence base for embryo selection strategies. This tripartite structure mirrors the intellectual framework identified through keyword co-occurrence and co-citation network analyses.

### Keyword co-occurrence analysis

**Figure 5:**
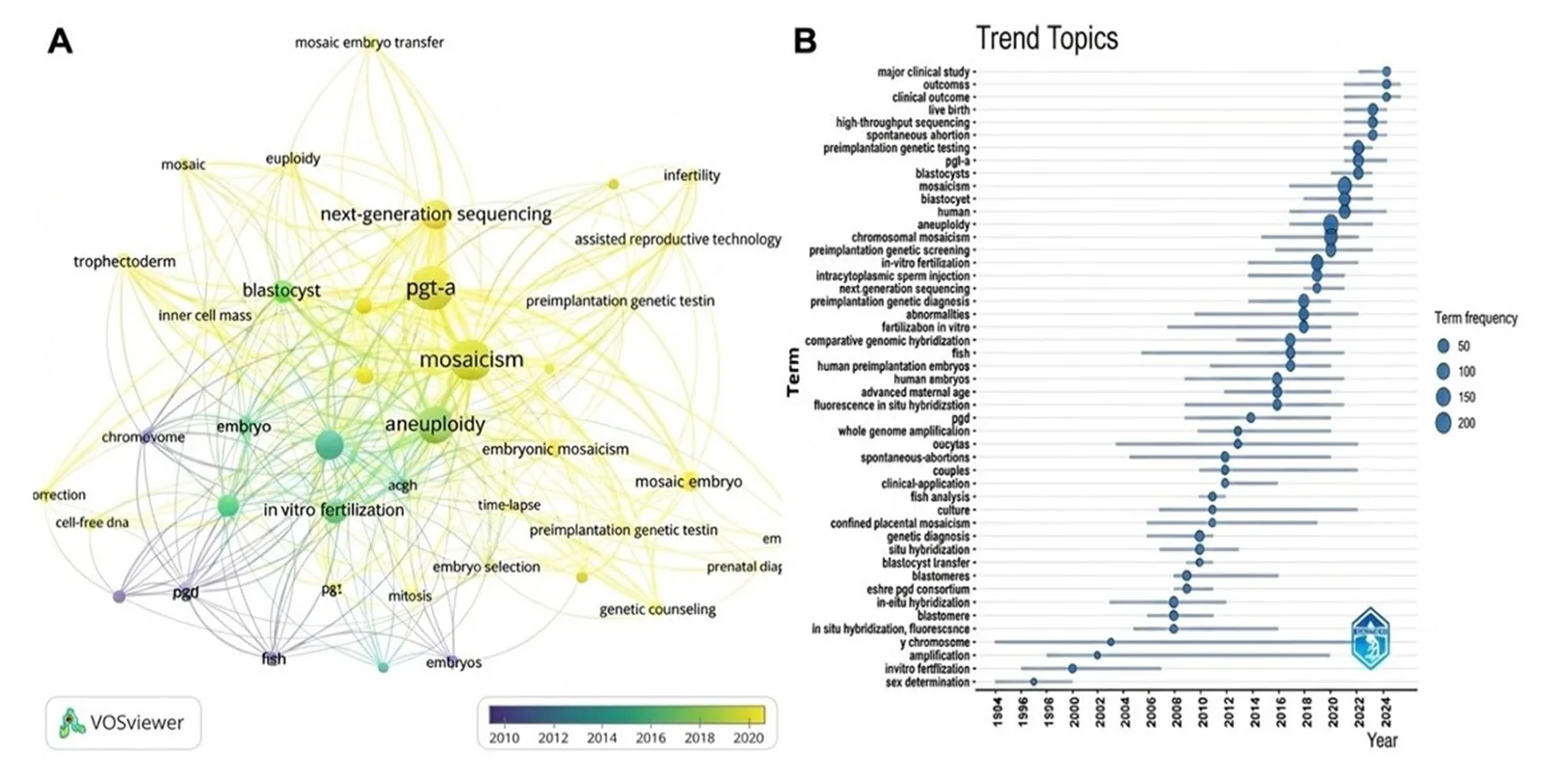
**(A)** Thematic evolution of research topics in embryonic mosaicism. Overlay visualization of keyword co-occurrence showing the evolution of research themes over time. Node colors represent the average publication year of associated keywords, illustrating the transition from cytogenetic studies toward genomic technologies and clinical outcomes. **(B)** Trend topics and frequencies over time. This trend topic analysis, derived from author keywords, maps the shifting focus of the scientific community. The timeline illustrates the transition from technical diagnostic validation (e.g., next-generation sequencing) to the clinical management and outcomes of mosaic embryos. The positioning and size of the nodes reflect the peak periods and relative intensity of research interest for each specific term. Panel 5A were generated using VOSviewer (v1.6.20). Panel 5B were generated using the Bibliometrix R package.

The temporal overlay visualization (Figure 5) reveals a clear directional shift in research focus across three chronological phases, each defined by a distinct technological and clinical context.

Early phase (1990–2010; *n* = 58 publications; mean citations/publication = 65.2): The keyword network of this phase was anchored by chromosomal mosaicism (*n* = 26), aneuploidy (*n* = 22), fluorescence *in situ* hybridization/FISH (*n* = 14), *in vitro* fertilization (*n* = 21), and preimplantation genetic diagnosis (*n* = 12). Research during this period was predominantly cytogenetic, focusing on detecting and quantifying chromosomal abnormalities in cleavage-stage embryos using FISH-based platforms. Outcome-oriented keywords—including live birth, implantation, and miscarriage—were largely absent from this phase, with implantation appearing in only three publications and live birth and miscarriage each occurring 0 times, reflecting the absence of intentional mosaic embryo transfer in clinical practice. The high mean citation rate (65.2 citations/publication) reflects the foundational status of early-phase studies, which continue to be cited as methodological and conceptual reference points.

Middle phase (2011–2017; *n* = 58 publications; mean citations/publication = 71.4): This phase is characterized by a technology-driven expansion of the keyword network. Aneuploidy (*n* = 35) and chromosomal mosaicism (*n* = 29) retained their central positions, while comparative genomic hybridization / aCGH (*n* = 18), array-CGH (*n* = 7), blastocyst biopsy (*n* = 6), trophectoderm (*n* = 7), and inner cell mass (*n* = 6) emerged as significant new nodes. Next-generation sequencing appeared with four keyword occurrences, marking the beginning of the NGS transition. Clinical outcome keywords remained nascent—live birth appeared in only 3 publications and implantation in 5—consistent with the pre-transfer era of mosaic embryo research. The highest mean citation rate of any phase (71.4 citations/publication) reflects the landmark status of middle-phase systematic reviews and platform-validation studies.

Recent phase (2018–2025; *n* = 274 publications; mean citations/publication = 21.7): this phase accounts for 69.5% of the total dataset and is marked by a decisive shift toward clinical and translational research themes. Aneuploidy (*n* = 143), mosaicism (*n* = 121), and chromosomal mosaicism (*n* = 95) dominated the keyword network, while PGT-A (*n* = 51), next-generation sequencing (*n* = 40), trophectoderm (*n* = 46), trophectoderm biopsy (*n* = 28), and blastocyst (*n* = 59) consolidated as core methodological terms. Critically, clinical outcome keywords underwent a step-change in frequency: implantation increased from 5 (middle phase) to 58 occurrences, live birth from 3 to 33, and miscarriage from 0 to 10, reflecting the transition from diagnostic to outcome-focused research following the landmark Greco et al. (2) report. Emerging research frontiers were also represented: artificial intelligence and machine learning appeared for the first time (*n* = 6 and *n* = 3, respectively), cell-free DNA and blastocoele fluid—proxies for non-invasive PGT-A research—each appeared six times, and spent culture medium appeared in two publications. The lower mean citation rate (21.7 citations/publication) reflects the citation immaturity of recent publications rather than diminished scientific impact.

Sensitivity analysis across alternative thresholds demonstrated that the primary thematic structure of the keyword network was robust to threshold selection. Although a lower threshold (*n* ≥ 3) yielded a substantially larger network (94 items, eight clusters) and a higher threshold (*n* ≥ 10) produced a more condensed map (19 items, three clusters), the three core thematic domains—cytogenetic characterization, NGS-based genomic diagnostics, and clinical outcomes—remained consistently identifiable across all three configurations. The primary nodes of highest centrality (aneuploidy, chromosomal mosaicism, PGT-A, next-generation sequencing) appeared across all threshold settings, confirming that the field's principal intellectual structure is not an artifact of threshold choice.

### Most productive authors and author collaboration network

All h-index values reported in this section are calculated within the present dataset and reflect the number of a given author's publications that received at least that many citations within the retrieved corpus; they are not equivalent to, and should not be compared with, career h-index values derived from comprehensive database queries.

The author-level bibliometric analysis identified 9 researchers with ten or more attributable publications in the verified dataset, representing the core scientific leadership of this research domain (Table 2). Author disambiguation—conducted by cross-referencing Scopus Author IDs with institutional affiliation strings—resulted in the exclusion of three name strings (Chen C, Lee M, Xu Y) that were found to aggregate multiple distinct researchers; their publication counts could not be reliably attributed to individual authors. Among the verified authors, Rubio C (IVIRMA Global, Spain) led in publication volume with 13 contributions, followed by six authors who each contributed 11 publications: Wells D (University of Oxford), Munné S (Reprogenetics/IVIRMA), Griffin DK (University of Kent), Capalbo A (Igenomix/Juno Genetics), Grifo JA (New York University), Gleicher N (Center for Human Reproduction). Viotti M and Greco E each contributed 10 publications.

**Table 2:** Most productive authors in embryonic mosaicism and mosaic embryo transfer research (minimum 10 verified publications; *n* = 9), ranked by publication count.

Rank	Author	Primary affiliation	Publications (n)	Citations (n)	H-index
1	Rubio, C	IVIRMA Global, Spain	13	816	11
2	Wells, D	University of Oxford, UK	11	1,485	11
3	Munné, S	Reprogenetics/IVIRMA, USA	11	1,393	11
4	Griffin, DK	University of Kent, School of Biosciences, Canterbury, UK	11	978	8
5	Capalbo, A	Igenomix/Juno Genetics, Italy	11	746	7
6	Grifo, JA	New York University, USA	11	656	9
7	Gleicher, N	Center for Human Reproduction, USA	11	508	10
8	Viotti, M	Zouves Foundation/IVIRMA, USA	10	734	8
9	Greco, E	UniCamillus International University/Villa Mafalda, Rome, Italy	10	574	7

Publication counts and citation metrics derived from the merged WoSCC–Scopus corpus (*n* = 394). Author disambiguation was performed by cross-referencing Scopus Author IDs and institutional affiliation strings; records that could not be unambiguously attributed to a single researcher were excluded. *H*-index values reflect within-dataset citation counts and are not equivalent to career *H*-index values. Primary affiliations reflect the most frequently recorded institution across each author's verified contributions.

Citation impact analysis reveals a distinct hierarchy compared to publication volume rankings. Wells D leads in total citations (1,485; *h* = 11), driven by landmark systematic reviews and foundational aCGH-based embryo studies. Munné S ranks second (1,393 citations; *h* = 11), reflecting seminal contributions spanning FISH-era mosaicism detection through the NGS and mosaic embryo transfer era. Griffin DK ranks third in total citations (978; *h* = 8), with contributions centered on chromosome segregation mechanisms and cytogenomic characterization of preimplantation embryos. Rubio C (816 citations; *h* = 11) and Capalbo A (746 citations; *h* = 7) complete the five highest-cited authors, reflecting the sustained influence of IVIRMA and Igenomix networks on the field.

Among the nine verified authors, geographic representation spans two principal regions. North American centers are represented by Munné S (Reprogenetics/IVIRMA, USA), Grifo JA (New York University, USA), Gleicher N (Center for Human Reproduction, USA), and Viotti M (Zouves Foundation/IVIRMA, USA). European contributions are led by Wells D and Griffin DK (University of Oxford and University of Kent, respectively, UK), Rubio C (IVIRMA Global, Spain), Capalbo A (Igenomix/Juno Genetics, Italy), and Greco E (UniCamillus International University/Villa Mafalda, Rome, Italy). The absence of verified East Asian authors from the minimum-threshold group should not be interpreted as diminished regional productivity; rather, it reflects the limitation of name-based author disambiguation for common East Asian name forms, a constraint explicitly acknowledged in the limitations section.

**Figure 6:**
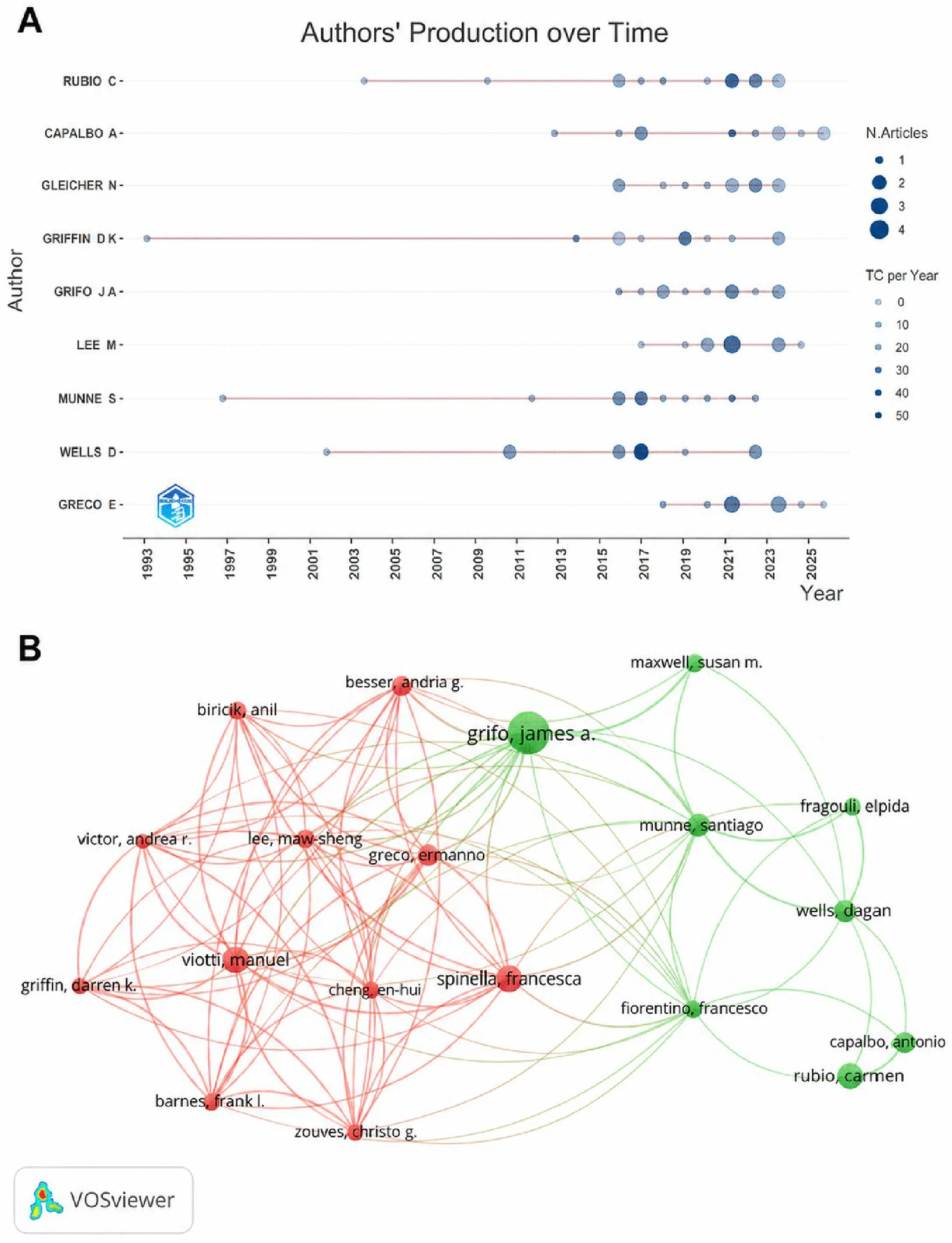
**(A)** Top Authors' annual publications over time. **(B)** Author collaboration network in embryonic mosaicism and mosaic embryo research. Co-authorship network generated using VOSviewer. Node size corresponds to publication count; link thickness reflects collaboration strength. Panel 6A were generated using the Bibliometrix R package. Panel 6B were generated using VOSviewer (v1.6.20).

Co-authorship network analysis (Figure 6) revealed a highly collaborative research community organized into three principal clusters: (1) a North American cluster centered on New York University, the Center for Human Reproduction, and the Zouves Foundation; (2) a Southern European cluster connecting IVIRMA Global, Igenomix, Institut Bernabeu, and Genoma Group; and (3) a Northern European cluster linking the University of Oxford, University of Kent, and Dutch reproductive genetics centers. Strong inter-cluster ties, particularly between the US and Southern European groups, reflect established collaborative relationships in multicenter clinical outcome studies and genomic platform validation efforts.

### Commercial affiliations among leading contributors

A notable structural feature of this research domain is the involvement of investigators affiliated with commercial providers of PGT-A and reproductive genetic testing services. Among the nine most productive authors, several had current or prior founding, executive, employment, or collaborative relationships with commercial genetic testing or IVF network organizations. Under the narrower declared-funding indicator defined in the Methods, explicit commercial funding was declared in only eight of 394 records (2.0%), indicating that industry involvement was more commonly reflected through author affiliation or employment than through declared study funding; the broader affiliation-based indicator is reported in the Discussion. These observations are intended as a descriptive characterization of the field's authorship structure, not as an assessment of individual investigators or study validity. A summary of commercial affiliations is provided in Supplementary Table S5.

### Co-citation and bibliographic coupling analysis

**Figure 7:**
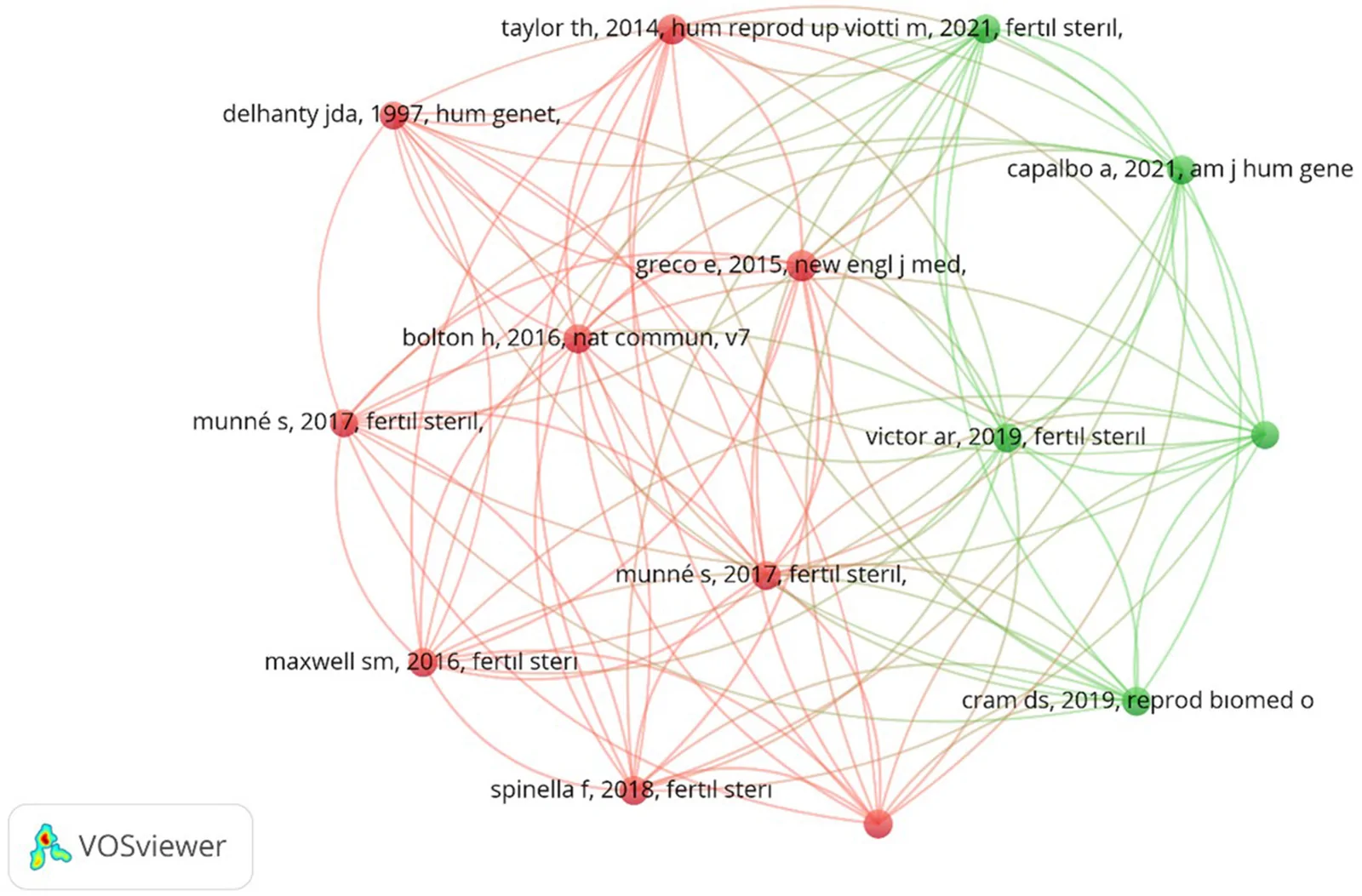
Co-citation network of influential publications in embryonic mosaicism research. Nodes represent cited references; node size reflects citation count. Links indicate frequent co-citation. Cited references with at least 50 citations were included. This figure were generated using VOSviewer (v1.6.20).

Co-citation analysis was performed on references with at least 50 citations, yielding three principal clusters that identify the intellectual core of the field (Figure 7). The most densely connected co-citation cluster comprised foundational diagnostic studies—including Munné et al. (1997), (7), Fragouli et al. (2011, 2017), and Delhanty et al. (1997)—reflecting the centrality of chromosomal characterization across successive detection platforms (FISH → aCGH → NGS). A second cluster grouped clinical outcome studies, anchored by Greco et al. (2) and Mastenbroek et al. (2011), representing the transition from diagnostic validation to evidence-based transfer decisions. The co-citation network thus mirrors the three-trajectory structure identified in the analysis of highly cited publications.

**Figure 8:**
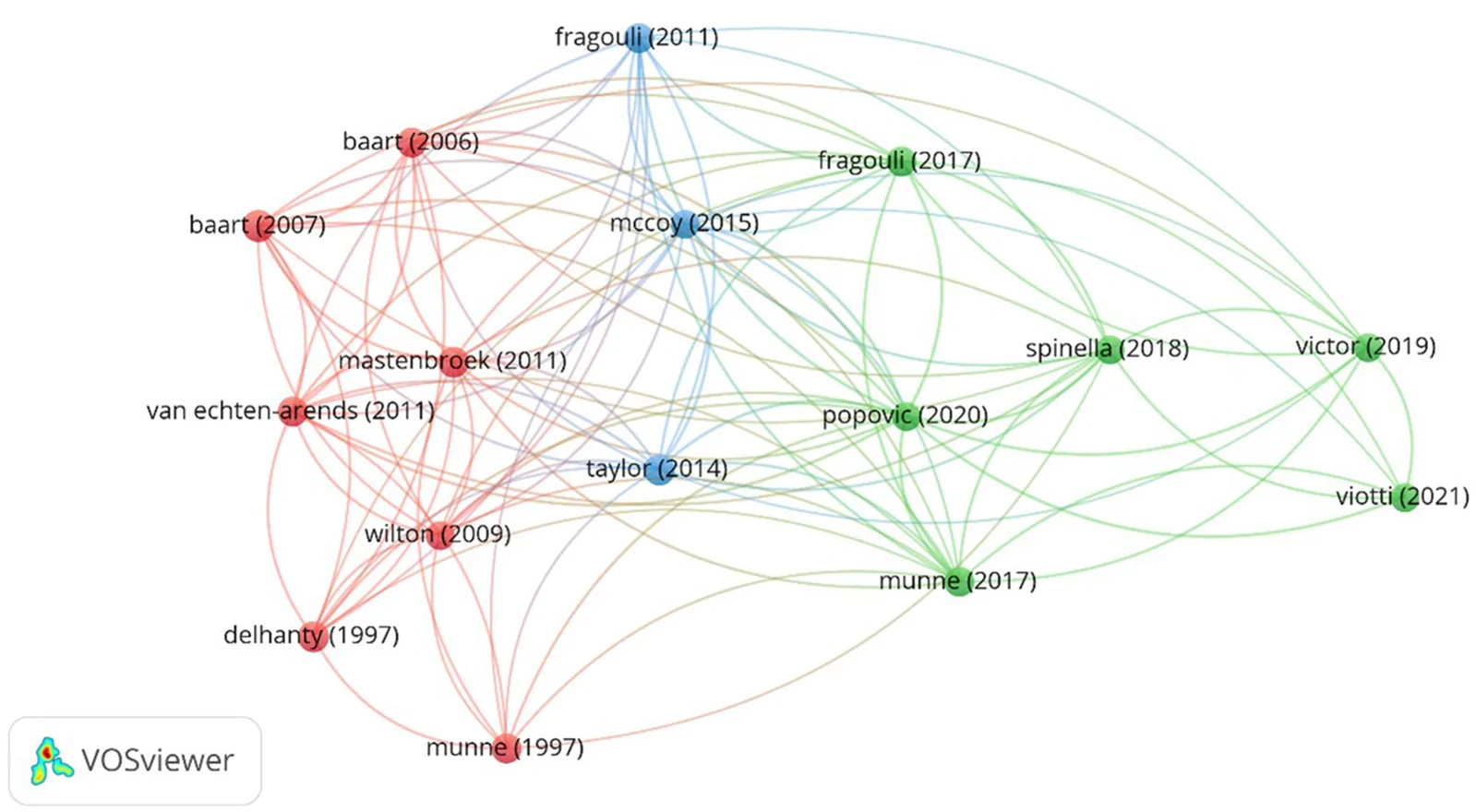
Bibliographic coupling network of publications on embryonic mosaicism. Network map illustrating relationships between publications based on shared references. Node size reflects citation impact; colors represent thematic clusters. This figure were generated using VOSviewer (v1.6.20).

Bibliographic coupling (Figure 8) identified three principal research clusters based on shared reference profiles: (1) a genomic diagnostics cluster focused on NGS-based mosaicism detection and copy-number quantification; (2) a clinical outcomes cluster examining implantation rates, miscarriage, and live birth following mosaic embryo transfer; and (3) an embryological mechanisms cluster investigating post-zygotic mitotic errors, trophectoderm–inner cell mass discordance, and self-correction pathways. The coupling strength between clusters (1) and (2) was notably high, indicating that genomic diagnostic studies and clinical outcome research share a substantial common reference base—consistent with the field's translational trajectory.

## Discussion

This bibliometric and science-mapping study provides a structured overview of global research on embryonic mosaicism and mosaic embryo transfer, revealing a marked acceleration in scientific output over the past decade. The thematic evolution identified here reflects a fundamental shift: from early cytogenetic characterization of chromosomal abnormalities toward a clinically integrated discipline that directly informs embryo selection strategies, patient counseling, and reproductive outcomes in modern IVF practice. Across all analytical layers—keyword co-occurrence, co-citation, and bibliographic coupling—three thematic domains emerge consistently: genomic diagnostics, clinical outcomes, and embryological mechanisms. The most recent keyword cohort (2022–2025) signals the emergence of a fourth domain encompassing artificial intelligence, machine learning, and non-invasive PGT-A. Throughout the following discussion, it is important to distinguish conclusions that follow directly from the bibliometric data—namely, publication volume, citation impact, collaboration structure, and the temporal evolution of research themes—from the broader, literature-based interpretations of biological mechanisms and clinical management that are provided here to contextualize those findings. Bibliometric analyses map the structure and trajectory of a research field; they cannot by themselves establish clinical efficacy or safety. Accordingly, any statements below regarding embryo selection, counseling, or clinical practice should be read as syntheses of the cited primary literature rather than as evidence generated by the present analysis, and clinical recommendations are framed with corresponding caution.

### Technological transformation as a driver of research growth

As quantified in the Results, publication output rose steadily and in close temporal association with NGS-related output, consistent with NGS adoption as a principal driver of the field's expansion rather than an incidental correlate. Unlike earlier platforms (FISH, array CGH), NGS quantifies copy-number variations, reclassifying embryos into meaningful mosaic categories and turning mosaicism into an actionable clinical factor. The subsequent rise in publications from 2021 to 2023 coincides with the dissemination of updated professional society recommendations. The PGDIS 2021 position statement (4), the ASRM committee opinion (6), and the ESHRE survey findings (5) collectively operationalized a risk-stratified framework for mosaic embryo management, which appears to have directly stimulated comparative outcome studies, systematic reviews, and meta-analyses within the field.

### Biological mechanisms and their clinical relevance

Embryonic mosaicism results from post-zygotic mitotic errors—such as nondisjunction, anaphase lag, and spindle abnormalities—during early cleavage divisions, when maternal transcripts primarily control embryonic cell-cycle checkpoints. This explains two key clinical observations: mosaicism rates do not follow the maternal-age pattern seen with meiotic errors, and some mosaic embryos can develop normally through self-correction mechanisms, such as the apoptotic elimination of aneuploid cells and the proliferation of euploid lineages (1, 8–11). The experimental basis for this self-correction model was substantially strengthened by Bolton et al. (2016)—the second most-cited publication in this dataset—whose mouse model demonstrated that aneuploid cells are not randomly distributed across embryonic lineages but are selectively depleted from the inner cell mass and preferentially retained in the trophectoderm. This lineage-specific elimination suggests that the developmental potential of a mosaic embryo is determined not merely by the proportion of aneuploid cells, but by their spatial distribution across ICM and trophectoderm compartments at the time of PGT-A biopsy—a biological insight with direct implications for how trophectoderm-based NGS results should be interpreted clinically.

These mechanistic findings support a probabilistic rather than binary clinical framework for mosaic embryo assessment. Registry-level outcome data indicate that low-level mosaicism (< 50%) is associated with reproductive outcomes approaching those of euploid embryos. In contrast, high-level mosaicism (>50%) and multichromosomal involvement are associated with significantly reduced implantation rates and elevated miscarriage risk (9, 12, 13). Crucially, this continuum-based model implies that the clinical significance of a mosaic PGT-A result cannot be fully determined from the copy-number percentage alone; chromosome identity, segmental vs. whole-chromosome involvement, and the known imprinting status of the affected region must all be considered in individualized counseling. The bibliometric trend toward increasing keyword frequency for “implantation,” “live birth,” and “miscarriage” in the recent phase (2018–2025)—rising from near-zero in the early and middle phases—reflects the field's gradual operationalization of this probabilistic framework into clinically actionable embryo selection protocols.

### Implications for obstetric management and prenatal medicine

The bibliometric trends identified in this study highlight areas of increasing relevance to obstetric and prenatal medicine. The emergence of keywords related to placental mosaicism, prenatal diagnosis, and neonatal outcomes in the 2020–2025 cohort reflects a growing recognition that the clinical consequences of embryonic mosaicism extend well beyond implantation. Confined placental mosaicism—in which chromosomal abnormalities are restricted to placental tissue—has been documented following mosaic embryo transfer and may contribute to adverse obstetric outcomes, including intrauterine growth restriction, preeclampsia, and unexplained pregnancy loss (8, 14). These findings highlight the need for prospective registry-based studies to determine whether pregnancies following mosaic embryo transfer require tailored antenatal surveillance, including serial fetal growth assessments and closer monitoring for uteroplacental insufficiency.

A clinically unresolved question concerns the appropriate use of invasive prenatal diagnosis following mosaic embryo transfer. Current professional society recommendations—including those of PGDIS (4), ASRM (6), and ESHRE (5)—acknowledge the option of prenatal confirmation but do not specify standardized indications or preferred modalities. The choice between chorionic villus sampling and amniocentesis carries distinct implications: CVS-based results may reflect placental rather than fetal karyotype, potentially overestimating fetal chromosomal abnormality in the presence of confined placental mosaicism, whereas amniocentesis provides a more direct assessment of fetal cell lines. Additionally, the risk of uniparental disomy (UPD)—which may arise through trisomy rescue in mosaic embryos—has received limited systematic investigation in the post-transfer literature. UPD involving imprinted chromosomal regions can have specific phenotypic consequences (e.g., Prader-Willi, Angelman, and Beckwith-Wiedemann syndromes) that may not be detected by standard chromosomal analysis alone. Prospective registries that capture cytogenetic, obstetric, and neonatal outcomes following mosaic embryo transfer are essential to address these gaps.

### Clinical translation and IVF decision-making

Registry-level data indicate that carefully selected mosaic embryos can lead to healthy live births without a demonstrable increase in chromosomal syndromes or major congenital anomalies (14). This principle has been extended to mosaic embryos identified in PGT-SR cycles, for which retrospective data suggest transfer outcomes comparable to those observed in standard PGT-A contexts (15). Nonetheless, implantation failure and miscarriage rates remain significantly higher than after euploid embryo transfer, reinforcing the principle that euploid embryos should be prioritized whenever available (4, 13, 16). Mosaic embryos are appropriately considered secondary candidates, with transfer decisions individualized based on the level of mosaicism, the chromosome involved, patient prognosis, and the cumulative availability of euploid alternatives (6, 10, 14, 21).

An important and unresolved controversy concerns the clinical significance of low-level mosaic signals detected by NGS-based PGT-A. Several authors have argued that intermediate copy-number values in this range may reflect technical noise or biological sampling variability rather than true embryonic mosaicism—a possibility with direct implications for patient counseling and embryo utilization (12, 17, 22). This debate underscores the urgent need for standardized reporting thresholds and validated risk-stratification models across platforms and laboratories.

### Commercial interests and research integrity

The bibliometric structure of this field cannot be fully interpreted without acknowledging the substantial commercial interests embedded within it. As shown above, industry-affiliated publications (classified as commercially linked according to the affiliation, funding, or conflict-of-interest criteria defined in the Methods) constitute about one-tenth of the corpus (39 of 394 records; 9.9%) yet account for a disproportionate share of its citation impact (14.7% of all citations; mean 52.2 vs. 33.4 citations per publication for the remainder). Commercial affiliation is nonetheless a minority feature even among the most influential work: three of the 15 most-cited publications (20%) carry such a link. This concentration is relevant to how the evidence base should be read: laboratories and investigators with a financial stake in the expanded clinical use of PGT-A may reasonably be expected to influence which questions are prioritized, how thresholds for reporting mosaicism are defined, and how favorably mosaic-embryo transfer outcomes are framed. It argues for interpreting effect sizes, threshold recommendations, and clinical-utility claims with attention to their funding provenance, and it strengthens the case for independent, non-industry replication, prospective registries, and standardized reporting frameworks maintained by professional societies rather than by individual commercial laboratories. These observations do not invalidate the scientific contribution of industry-affiliated research; indeed, much of the foundational and methodological work in this field has come from these same groups. They do, however, highlight the need for transparent conflict-of-interest reporting, independent validation, and cautious interpretation of clinical-utility claims in a field in which diagnostic technologies carry direct commercial value.

### Emerging technologies and future directions

The emerging keyword cluster encompassing artificial intelligence, machine learning, and non-invasive PGT-A—though modest in current bibliometric weight—points toward a likely methodological frontier in mosaic embryo research. Non-invasive PGT-A, which analyzes cell-free DNA shed into the embryo culture medium, offers the theoretical advantage of sampling genetic material from both the trophectoderm and the inner cell mass without the procedural risks of biopsy (18). Current limitations—including low DNA input, the risk of maternal contamination, and variable concordance with invasive biopsy results—are increasingly being addressed through deep learning-based signal-discrimination frameworks such as DECENT, which reconstructs embryonic copy-number variation profiles from maternally contaminated spent culture medium samples (19), and multimodal assessment platforms integrating time-lapse morphokinetics, metabolomic profiling, and genomic datasets (20).

The transition from a binary euploid/aneuploid classification toward AI-supported probabilistic embryo competence models is an emerging trajectory identified in the recent literature. However, the current bibliometric signal remains modest in absolute terms; the directional consistency of this trend across the 2022–2025 cohort suggests it merits prospective investigation in future studies. These directions—particularly artificial-intelligence-based embryo competence modeling and non-invasive PGT-A—should be regarded as emerging research avenues rather than established or validated clinical trends. Their current bibliometric weight is small, the supporting clinical evidence remains preliminary, and premature clinical adoption ahead of adequately powered, independent validation would be inappropriate.

### Strengths and limitations

This study offers one of the most comprehensive science-mapping evaluations of mosaic embryo research to date, combining publication trend analysis, citation impact assessment, and multi-layered network visualization to characterize both the intellectual structure and collaborative architecture of the field. Although the primary analytical corpus was constructed from WoSCC and Scopus, PubMed-unique records were excluded from the network and citation-based analyses due to metadata incompatibility. This may have excluded a small number of otherwise relevant publications, although validation of PubMed coverage indicated high overlap with the primary corpus.

Author-level bibliometric counts are subject to name disambiguation uncertainty, particularly for common East Asian name forms. In the present dataset, the name strings “Chen C,” “Lee M,” and “Xu Y” were each found to aggregate multiple distinct researchers, confirmed by the presence of two or more distinct Scopus Author IDs or mutually exclusive institutional affiliations within a single name string. These three entries were therefore excluded from Table 2. Future bibliometric studies in this domain would benefit from ORCID-based author identification, thereby eliminating disambiguation uncertainty.

Country-level contribution estimates varied according to the analytical unit used: all-author country assignment identified 48 contributing countries, whereas corresponding-author assignment, used for Supplementary Table S2, identified 34 countries. Both figures are reported to ensure transparency. Citation-based indicators should be interpreted with caution because recent publications, particularly those from 2024 and 2025, have had limited time to accrue citations. Additional limitations include the exclusion of gray literature sources, such as ClinicalTrials.gov, PGDIS, ESHRE conference proceedings, and reproductive medicine preprint repositories, as well as the absence of ORCID-based author disambiguation, which may affect the precision of author-level productivity estimates.

The search strategy also has inherent constraints. Although the Boolean query was designed to capture the core terminology of the field, it did not explicitly include several increasingly used descriptors, such as segmental, complex, high-level, and low-level mosaicism. These terms are likely to co-occur with broader anchor terms such as “chromosomal mosaicism,” “mosaic embryo,” and “mosaic blastocyst,” but a small number of narrowly indexed publications may have been missed. Future analyses would benefit from a more granular mosaicism-specific search strategy. Finally, the protocol was not prospectively registered. Because bibliometric theme selection, cluster labeling, and narrative interpretation involve some degree of author judgment, the absence of protocol registration should be considered a potential source of interpretive subjectivity. Relatedly, the assessment of commercial affiliation and funding relied on the affiliation, funding, and conflict-of-interest information disclosed within the publications themselves; undisclosed commercial relationships could not be captured, and the reported proportions should therefore be regarded as conservative lower-bound estimates of the true extent of industry involvement.

## Conclusion

This bibliometric and science-mapping analysis of 394 publications spanning 36 years (1990–2025) confirms that research on embryonic mosaicism has undergone a fundamental transformation—from early cytogenetic characterization to a clinically integrated discipline directly informing embryo selection strategies, patient counseling, and IVF decision-making. The marked acceleration in annual output following 2015, coinciding with the widespread adoption of NGS-based PGT-A and the subsequent dissemination of major professional society guidelines (PGDIS 2021, ESHRE 2022, ASRM 2023), reflects both technological and institutional drivers of this growth. The United States and a small number of specialized European centers have led this expansion, with international collaborative networks emerging as a defining structural feature of the field.

Despite these advances, three questions continue to define the field's unresolved research agenda: the mosaicism level at which transfer risk becomes clinically unacceptable, the biological mechanisms underlying *in vivo* self-correction, and the long-term health outcomes of children born following mosaic embryo transfer. Addressing these gaps will require carefully designed prospective registries, standardized reporting frameworks, and sustained interdisciplinary collaboration across reproductive medicine, obstetrics, and clinical genetics. Emerging methodological frontiers—including AI-assisted embryo competence modeling and non-invasive PGT-A—represent the next phase of translational research in this rapidly evolving field. These findings provide a structured science-mapping foundation that may inform future research planning, evidence synthesis, and the development of standardized reporting frameworks in assisted reproductive technologies.

## Data Availability

The original contributions presented in the study are included in the article/Supplementary material, further inquiries can be directed to the corresponding author.
